# Low throughput screening in neuroscience: using light to study central synapses one at a time

**DOI:** 10.1117/1.NPh.10.4.044407

**Published:** 2023-10-24

**Authors:** Léa Caya-Bissonnette, Jean-Claude Béïque

**Affiliations:** University of Ottawa’s Brain and Mind Research Institute and Centre of Neural Dynamics, Ottawa, Ontario, Canada

**Keywords:** two-photon uncaging, synapse, plasticity, α-amino-3-hydroxy-5-methyl-4-isoxazolepropionic acid receptor, N-methyl-D-aspartate receptor, dendrites

## Abstract

Neurophotonic approaches have fostered substantial progress in our understanding of the brain by providing an assortment of means to either monitor or manipulate neural processes. Among these approaches, the development of two-photon uncaging provides a useful and flexible approach to manipulate the activity of individual synapses. In this short piece, we explore how this technique has emerged at the intersection of chemistry, optics, and electrophysiology to enable spatially and temporally precise photoactivation for studying functional aspects of synaptic transmission and dendritic integration. We discuss advantages and limitations of this approach, focusing on our efforts to study several functional aspects of glutamate receptors using uncaging of glutamate. Among other advancements, this approach has contributed to further our understanding of the subcellular regulation, trafficking, and biophysical features of glutamate receptors (e.g., desensitization and silent synapse regulation), the dynamics of spine calcium, and the integrative features of dendrites, and how these functions are altered by several forms of plasticity.

Scientists need to be many things, including pedagogues. In doing so, they often rely on intuitive and historically prominent imagery to capture the imagination of their audience (and their own) and to convey a sense of meaning and importance. For instance, in this exercise, many cellular neuroscientists will readily show the drawings of neurons by Ramón y Cajal,^1^ which instill a profound sense of wonder and puzzlement, still, more than 100 years later. Other cellular neuroscientists, those with perhaps a more abstract mind, will rather intuitively converge to the iconic voltage traces of the action potential from Hodgkin and Huxley as the expression of the elemental essence of the brain in action. Others may think of the banal looking blips analyzed by Sir Bernard Katz and colleagues in the 1950s, which established the foundation of how we still conceptualize information transfer at synapses. Yet, for decades, the experimental approaches of cellular electrophysiology and imaging were largely progressing in parallel, with little direct cross-pollination. The development of multiphoton microscopy, along with key progress in synthetic chemistry, has coalesced these disciplines wherein experimenters can observe and manipulate neural processes simultaneously. By allowing the activation of individual brain synapses with exquisite spatial and temporal control, two-photon (2P) uncaging of caged molecules provides a compelling illustration of substantial progress in our understanding of neural function afforded by the still expanding field of neurophotonics.

## 1 Where Chemistry, Optics, and Electrophysiology Collide

At their core, individual neurons are analog-to-digital converters. They harbor at times tens of thousands of individual synapses and continuously integrate the tiny electrical synaptic events by means of a complex mixture of linear and non-linear operations to render a digital decision: a spike. Yet, any student of the brain knows that synaptic transmission is partly a chemical process, offering corollary practical experimental opportunities. Indeed, it is fairly straightforward to synthesize neurotransmitters and several methods have been developed over the years to provide means for their local and rapid application to study synaptic mimicry. However, these methods (e.g., iontophoresis) inherently present spatial and/or temporal limitations. The development of photolabile “caged” molecules, combined with the relative ease of controlling light in space and time, offers a powerful approach to (at least partly) address these limitations. A biologically active molecule is said to be caged when it is made inert by the covalent bond of a photochemical group.^2^ Kaplan et al.^3^ synthesized the first caged compound, caged adenosine triphosphate (ATP). Photolysis of the caged ATP compound (or “release” of the active molecule) was induced with near-ultraviolet light and was instrumental in elucidating important functional aspects of the ${\mathrm{Na}}^{+}/K^{+}$ ATPase pump. In the next decade, the first caged neurotransmitters, including for glutamate, the primary excitatory neurotransmitter in the brain, were developed by the Hess group.^4^^–^^7^ Although the use of these photoactivated caged compounds were useful for molecular biology studies, several of their features made them suboptimal for direct neuroscience applications.

**Fig. 1:**
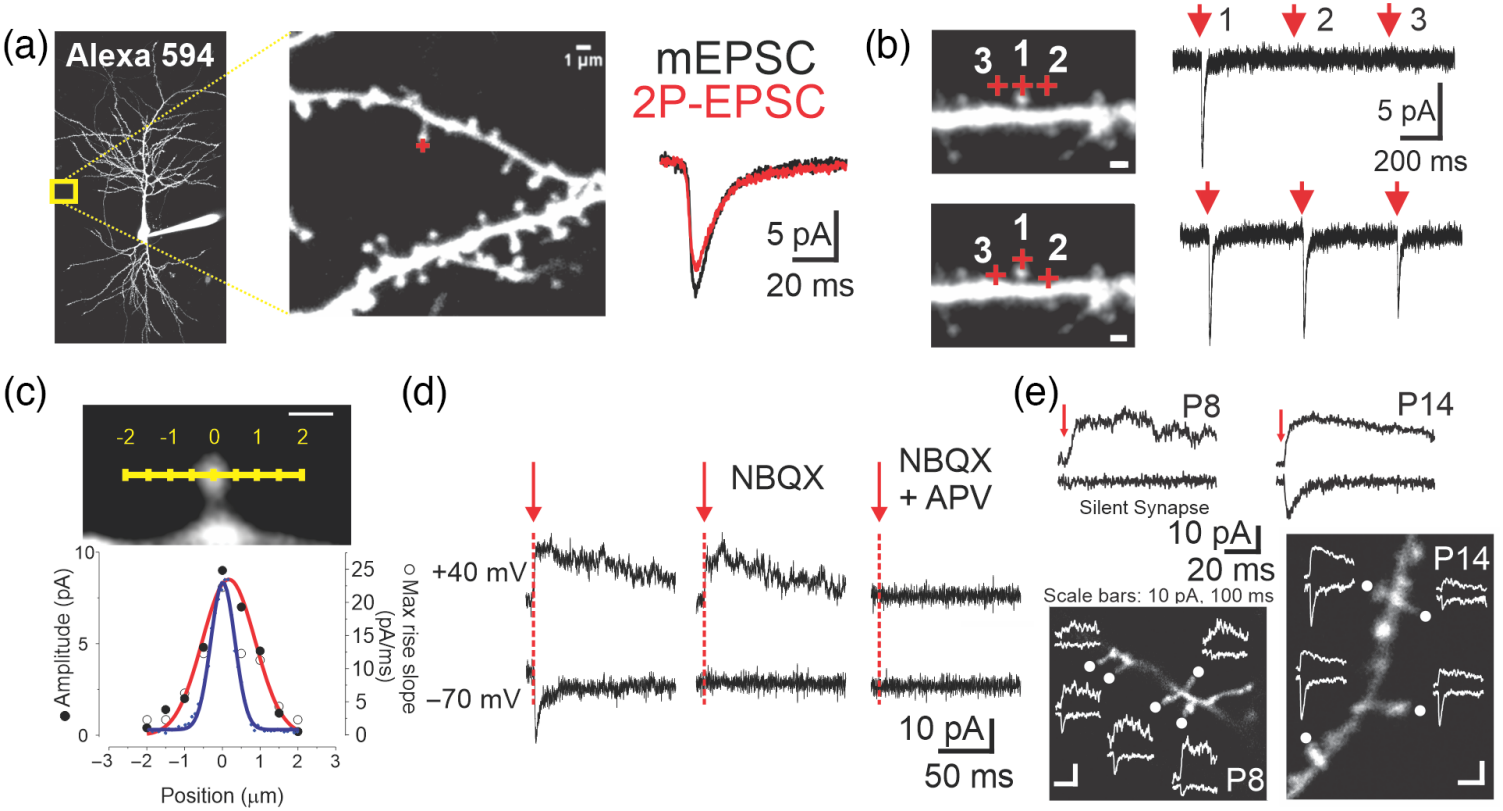
2P MNI-glutamate uncaging. (a) 2P EPSCs were induced by brief laser illuminations onto a single spine (denoted with the red cross hair) from a CA1 neurons in a hippocampal slice. The neuron was filled with Alexa 594 to outline neuronal morphology (spines, dendrites; note the recording electrode extending from the soma). The light-evoked EPSCs exhibit similar temporal dynamics (rise and decay) to those of EPSCs induced by endogenously released glutamate (mEPSC, miniature EPSCs). (b) Glutamate uncaging induced on a spine or from varying distances from a dendritic surface (top versus bottom panel) provides an estimate of the effective resolution of the uncaging process. (c) A similar experiment was carried out, but from uncaging along the orthogonal axis of a dendritic spine. The amplitude (red trace) and max rise slope of the evoked 2P EPSC is shown in the bottom panel, overlayed on the fluorescence profile of the spine (blue trace). (d) 2P uncaging of MNI-Glu induced the activation of glutamate receptors of both the AMPA and NMDA subtypes, blocked by NBQX and APV, respectively. (e) Glutamate uncaging detects developmentally regulated silent synapses, that is, synapses that harbor NMDARs, but no AMPARs. This method is amenable to determine the spatial distribution of synapses of different weights along dendritic arbors. Panels (a) and (c) reproduced from Ref. 11 with permission; panels (b) and (e) reproduced from Ref. 12 with permission from Elsevier.

A common limitation of one photon (1P) microscopy lies in the scattering of light in tissue, such as the brain (and resulting degradation of the associated diffraction limited spot). For neurotransmitter uncaging purposes, this limitation is further compounded by the fact that the uncaging event *per se* is not restricted to the focal plane, but rather occurs along the entire length of the excitation light path in the sample. Although multiphoton microscopy not only improves the spot quality for imaging in tissue, it also enables the uncaging event to be limited to a volume roughly that of a diffraction-limited spot and, therefore, approximating the point-source diffusion of endogenous vesicle release at synapses *in situ*. Yet, the full realization of this approach could only be realized with the development of caged molecules exhibiting a favorable 2P cross section, along with a broad set of parallel advantageous functional and practical features (stability, quantum yield, receptor affinity, and others). For synaptic physiology purposes, an important milestone was achieved with the development of MNI-glutamate^8^^–^^10^ that allowed the uncaging of glutamate onto visually identified synaptic spines to trigger excitatory postsynaptic currents (EPSC) and potentials [Fig. 1(a)^11^^,^^13^^–^^15^].

## 2 Synaptic Function and Dendritic Integration

The hardware required to implement 2P imaging dovetails well with that of traditional cellular electrophysiology. Although the uncaging approach has been used for several neurotransmitters, we briefly here mention some of the advances that were derived from the use of MNI-glutamate. In a typical experiment (Fig. 1), neurons are recorded in the whole-cell configuration thereby allowing the dialysis of the intracellular compartment with fluorescent dyes in order to either delineate subcellular compartments (e.g., spines) or to monitor some dynamical process (e.g., intracellular calcium transients). A brief (0.5 to 1 ms) illumination with a Ti:saphire laser (tuned at ∼720  nm) achieves an effective focal release of glutamate with a roughly <2  μm (x,y) spot size [Refs. 11 and 13; Figs. 1(b) and 1(c)]. The experimenter calibrates the laser power such that the amplitude of synaptic currents closely matches that of those triggered by endogenously released glutamate (*ca.*, 5 to 20 pA). In these conditions, the effective volume of uncaging (which is a convolution of the size of the diffraction limited spot and diffusion) is greater than that resulting from the vesicular release of glutamate (Ref. 16 and unpublished observation) and needs to be carefully taken into account. Nonetheless, this process readily activates endogenous glutamate receptors of the α-amino-3-hydroxy-5-methyl-4-isoxazolepropionic acid (AMPA) and N-methyl-D-aspartate (NMDA) subtypes, exhibiting a kinetic profile that reasonably well approaches that of when these receptors are activated by release of endogenous glutamate [Fig. 1(d)].

By allowing unparalleled spatial and temporal control of glutamate receptor activation, this broad approach has been conducive to substantial progress. The sole ability to activate AMPA receptors (AMPARs) in a controlled and deterministic fashion provides a useful means to parameterize several of their biophysical features *in situ*. For instance, this overall approach contributed to the demonstration that the desensitization of AMPARs (occurring for instance during burst transmission) is at least partly offset by the lateral diffusion of these receptors along the surface of dendritic/synaptic membrane.^17^ The ability to activate glutamate receptors on different subcellular compartments of neurons (e.g., spine versus dendritic compartment) was also used to demonstrate that the homeostatic synaptic plasticity process differentially regulates the subcellular expression of AMPARs and NMDA receptors (NMDARs). As a last example, it is known that a subset of synapses are devoid of NMDARs and are therefore called silent synapses. Although their existence has been demonstrated using traditional electrical stimulation in slices,^18^^–^^20^ 2P uncaging experiments established that silent synapses were preferentially found on thin filopodia protrusions.^13^^,^^21^ Analogous experimental approaches were used to map the distribution of silent synapses onto developing dendritic arbor, revealing a striking clustered distribution that is believed to be a manifestation of cooperative plasticity rules [Fig. 1(e)^12^].

**Fig. 2:**
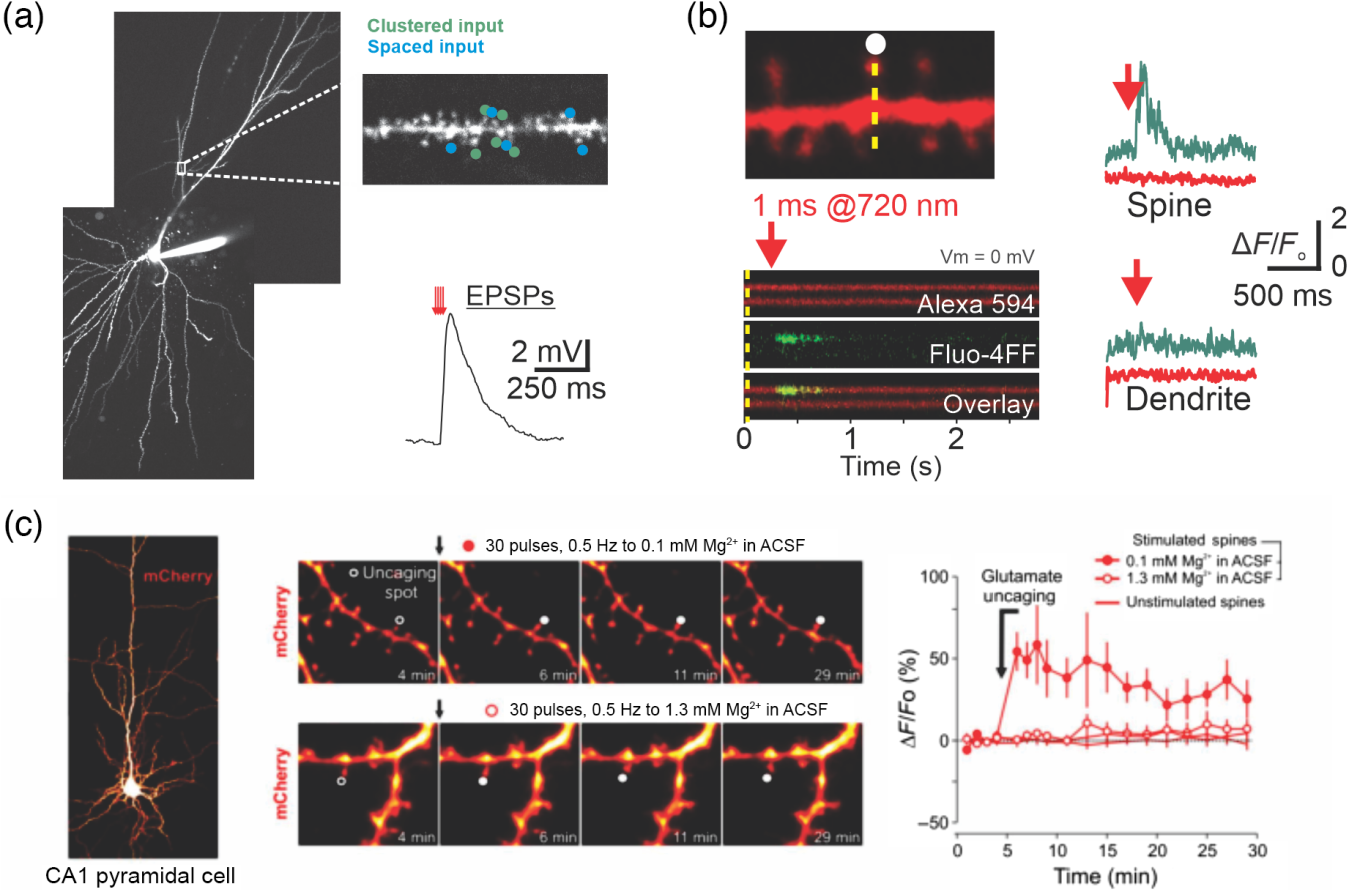
(a) 2P MNI-Glu uncaging can be done quasi-simultaneously on closely localized (i.e., clustered) spines using traditional galvo-based beam steering. (b) Continuous line scan acquisition over a region spanning both spine and dendrite (measured using a low affinity ${\mathrm{Ca}}^{2+}$ fluorescent dye, Fluo-4FF) shows ${\mathrm{Ca}}^{2+}$ dynamics in response to single spine uncaging. The ${\mathrm{Ca}}^{2+}$ signals reflect ${\mathrm{Ca}}^{2+}$ entry mainly through activation of NMDARs (not shown). (c) All-optical structural plasticity. A neuron expressing the fluorescent protein mCherry was targeted for single synapse uncaging. Repetitive uncaging onto a single spine in conditions favoring the opening of NMDARs ($0.1\;\mathrm{mM}\;{\mathrm{Mg}}^{2+}$; top middle panel) lead to a persistent spine enlargement (far right panel). Note that this structural plasticity in these conditions is spine-specific since closely localized spines do not exhibit shape alterations. Panel (c) reproduced from Ref. 24 with permission.

2P uncaging allows to test the impact of different spatial and temporal patterns of synaptic input by systematically positioning several uncaging spots along various length of dendritic segments. One area of research that has substantially benefited from such ability is dendritic integration. While a review of these contributions is beyond the scope of this paper, it is noteworthy that uncaging approaches have been central in providing key experimental confirmation of several non-linear dendritic operations that had been proposed by earlier computational approaches. As a mere example, influential work from the early 1990s proposed that clustered, as opposed to distributed, synaptic inputs would be subjected to non-linear amplification and thereby have a privileged influence on action potential output.^22^ The experimental confirmation that this class of dendritic operation occurred in neurons, along with mechanistic insights, was made possible by 2P uncaging approaches [Fig. 2(a); e.g., Refs. 23, 12, and 25–27). The combination of these experimental advances with modern computational approaches is beginning to uncover the catalog and full algorithmic role of these dendritic operations in controlling neural and network dynamics.

The study of spine behavior was further explored by 2P uncaging but paired with 2P calcium imaging [Fig. 2(b)]. Among other advances, these studies have shown the existence of calcium compartmentalization in spines,^28^ and how this behavior of calcium signals is regulated across postnatal development^12^ and during synaptic plasticity (e.g., behavioral timescale synaptic plasticity^29^).

## 3 Synaptic Plasticity

**Fig. 3:**
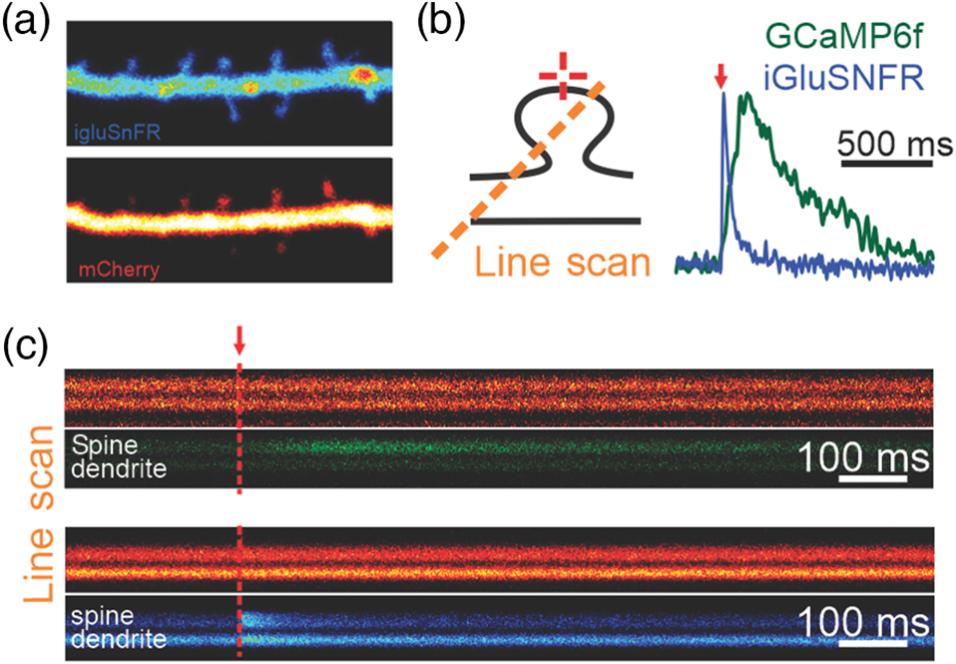
2P MNI-Glu uncaging used in combination with imaging of genetically encoded sensors. (a) Dendritic and spine expression of the glutamate sensor iGluSNFR expressed in a CA1 pyramidal neuron. (b) Local photolysis of glutamate by 2P uncaging triggered a rapid fluorescence signal from the glutamate sensor iGluSNFR. Parallel experiments showed a far slower response when NMDAR-dependent ${\mathrm{Ca}}^{2+}$ influx induced by MNI-Glu uncaging was monitored using the GCaMP6f sensor. (c) The same experiments as in B are plotted showing fluorescence of GCaMP6f (green) and iGluSNFR (blue) over time. The red vertical lines indicate the time of uncaging. Reproduced from Ref. 16 with permission.

Since Bliss and Lømo’s breakthrough experiments,^30^ the ability of synapses to undergo stable and long-term alterations constitutes the mainstay of current models of learning and memory. Traditionally, synaptic strength in slice experiments is monitored by electrically stimulating an unknown number of axons with largely undefined spatial location. Not surprisingly, 2P uncaging approaches have been used to study plasticity induction on identified spines [Fig. 2(c)] and directly demonstrated the existence of structural plasticity,^31^ where the volume of dendritic spines increased following plasticity induction along with other structural features.^32^ The ability to activate individual synapses has further been used to study molecular aspects of plasticity, for instance by investigating the effects of molecular manipulations on the expression of structural plasticity or surface expression of AMPARs (determined for example by the detection of Phluorin-tagged AMPAR subunits^33^). While in principle 2P uncaging approaches can be used to directly monitor the function of endogenous AMPARs following plasticity induction, these experiments are nonetheless highly challenging. Indeed, given the small volume of the uncaging spot, minute spatial drift over the time course of a typical synaptic plasticity experiments (i.e., >30  min) in principle may lead to high variability in the effective amount of glutamate reaching the spine under study, thereby introducing artifactual changes in EPSC amplitudes. To address this issue, Soares et al., in 2017,^24^ made use of the iGluSNFR, a genetically encoded fluorescent indicator of glutamate release (Fig. 3), to longitudinally monitor the amount of glutamate reaching the spine simultaneously with the magnitude of the EPSC induced by the photolysis event. This combined approach controlled for spatial drift and showed that endogenous AMPARs were rapidly trafficked to spines following plasticity induction.^24^

## 4 Conclusion

While one can be at once a disciple of Cajal and of the famed baseball legend Yogi Berra who pointed out that “Sometimes you can tell a lot about something just by watching,” simply looking is not always enough. How can we know how neurons, with their complex arborization and their odd-looking protuberances, such as the mysterious and initially controversial *espinas*, really work? The advent of 2P uncaging approaches allows us to manipulate the activity of visually identified single synapses with unmatched ease and precision. Of course, it is not perfect and brings its share of inevitable compromises. For instance, one would want more and smaller uncaging spots, along with far greater and flexible spatial and temporal control of a broad array of these activation spots, including for *in vivo* application.^34^ Yet, considering the progress made in neurophotonics in recent decades, we only need a bit of patience, as the future is bright.
